# Alcohol Consumption during the COVID-19 Lockdown Period: Predictors of At-Risk Drinking at Different AUDIT-C Cut-Off Thresholds

**DOI:** 10.3390/ijerph182413042

**Published:** 2021-12-10

**Authors:** John H. Foster, Colin R. Martin, Josh P. Davis

**Affiliations:** 1 School of Health Sciences, Institute for Lifecourse Development, University of Greenwich, London SE9 2UG, UK; 2 Institute for Health and Wellbeing, University of Suffolk, Ipswich IP4 1QJ, UK; C.Martin6@UOS.ac.uk; 3 School of Human Sciences, Institute for Lifecourse Development, University of Greenwich, London SE10 9LS, UK; j.p.davis@gre.ac.uk

**Keywords:** COVID, alcohol, home drinking, HDAS, purchasing

## Abstract

During the COVID-19 pandemic, alcohol consumption was largely confined to drinking in the home. There has been little research examining variables associated with risk in home drinking. The study employed an online survey of (*n* = 1128) individuals who had been recruited for their face recognition skills (*n* = 838, 70.9% females, mean age 45.05 (12.3 SD)). The main dependent variables were three different AUDIT-C cut-off scores for at-risk drinking: (a) 5 for both genders as recommended by Public Health England, (b) 7 for females and 8 for males (cut-off for students and young people) and (c) 8 for both genders (individuals seeking online help for their drinking). Among the independent variables were gender and age, motivations for home drinking using the Home Drinking Assessment Scale (HDAS), purchasing patterns, context of drinking and health and wellbeing. The predictors following hierarchical logistic regressions were for (a) purchasing alcohol online or at a supermarket and emotional HDAS scores, (b) purchasing alcohol online or at a supermarket and for parties, drinking alone and with other members of the household and emotional and practical reason HDAS scores, (c) as for b with the addition that men were more likely to be at-risk drinkers. At-risk drinking in the pandemic was explained by motivational reasons, purchasing patterns and situational factors.

## 1. Introduction

On 23 March 2020, the UK went into lockdown due to the COVID-19 pandemic. Pubs, bars and restaurants closed and re-opened on 4 July 2020. This provided a naturalistic opportunity to examine alcohol consumption in a domestic setting, predominantly at home. Stevely et al. (2021) [1] examined alcohol consumption during the early stages of the pandemic in England and Scotland. No change was observed in Scotland, suggesting that alcohol consumption at home had compensated for the pubs, etc., being closed. For England, there was a fall in consumption, indicating that the amount consumed at home was less than that consumed if the pubs had remained open. An online international survey of drinkers (*n* = 859) during the first COVID-19 lockdown (collected during approximately the same time period as the current study) found that 31% increased their drinking, but a greater proportion of respondents reported a reduction in their drinking, while the remainder reported no change [2]. A cross-European study [3] found that during the first months of the COVID-19 lockdown, alcohol consumption had also declined.

Economic evidence suggests a shift in beverage consumption during the initial months of the lockdown period. The Institute of Alcohol Studies (IAS) [4] in the UK reported that alcohol duty receipts from April to July 2021 were 2.4% lower than those from the same period in 2019–2020. Beer and cider receipts were reduced; in contrast, receipts for wine and spirits were increased. The IAS also reviewed eleven research papers that examined alcohol consumption during the pandemic and concluded that the general trend was to reinforce already existing drinking patterns. Below is a direct quote from their briefing:

“General population survey data indicate there has been a rise in the proportions of both non-drinkers and higher risk drinkers, and that similar proportions of people are drinking more than before and less than before”.

[4]

There has been an update from Public Health England [5]. When comparing data from 2019–2020, there was a 20% rise in alcohol-specific deaths, including a 15.4% rise in deaths due to alcohol poisoning. Deaths from alcoholic liver disease comprised 80.3% of all alcohol-related deaths, and there was a 20.8% increase in these from 2019 to 2020. This indicates that although for many alcohol consumption has been relatively stable over the lockdown periods, but some subgroups of heavy drinkers have been drinking at harmful, and at times fatal, levels. This disproportionate adverse impact of the lockdown was confirmed in a Norwegian study [6].

Bell and Britton [7], in a 20+ year longitudinal study of middle-aged adults [8], found that poor mental health was associated with heavy alcohol consumption in a nonclinical sample. A general population study conducted during the lockdown period discovered found an increase of anxiety and depression in the sample [9]. The vast majority of drinking during the lockdown period occurred at home.

There has been work that has considered the motivations underpinning home drinking. Qualitative findings [10] suggest these are cost, convenience and relaxation. These results were confirmed in a quantitative survey of University Staff (*n* = 488) [11], using the Home Drinking Assessment Scale (HDAS) [12], to measure motivations for drinking at home. The authors [11] aimed to measure alcohol-purchasing patterns and demographic factors to determine what factors might predict at-risk home drinking using the popular AUDIT survey tool [13]. The significant predictors were gender (females were more likely to be at-risk drinkers), younger age, more frequent drinking at home, preloading (drinking before going out), purchasing alcohol in an off-licence and financial cost. Using the same measures as in [11], Canfield et al. [14] surveyed a sample of home-drinking women 30+ (*n* = 411). On this occasion, the significant predictors of at-risk drinking were drinking at home every day, purchasing alcohol as part of weekly shopping and preferring to drink at home.

There is a shorter version of the AUDIT, this is the AUDIT-C [15], that uses the first three questions of the AUDIT to focus upon frequency and level of alcohol consumption. There is no universally agreed cut-off point for problem drinking using either the AUDIT or the AUDIT-C in the scientific community. Public Health England [16] suggest a cut-off score of 5 for both genders. This is consistent with the recent recommendation that sensible drinking in the UK should be at or below 14 units of alcohol per week with two drink-free days for both genders [17]. Subsequent to this recommendation, there has been work that suggests the cut-off for at-risk drinking for AUDIT-C in different groups should be higher. In a sample of (*n* = 5401) Dutch university students [18], a cut-off of 7 was recommended for female and 8 for male students. Further work assessing adults seeking online help (*n* = 3720) as a result of their alcohol consumption concluded that the AUDIT-C cut-off for both genders should be 8 [19].

This study examines predictors of at-risk drinking using different cut-off points in a general population sample who completed an online survey. The focus of the study is on reasons for drinking at home, alcohol purchasing patterns, sociodemographic factors and health and well-being. It provides greater data concerning the relationship between at-risk drinking and consumption of alcohol at home during the COVID-19 lockdown period.

## 2. Methods

Participants were invited from the University of Greenwich Face and Voice Recognition volunteer participant worldwide database (*n* = 48,000), mainly constructed to encourage participation in cognitive psychology projects. To be included, participants take a series of face recognition tests, and on completion, can optionally consent to be contacted for future research projects conducted by the current third author, his students or other staff at the university. The key defining feature of the database is that participants tend to have far better than average face and voice recognition ability, e.g., [20], but most possess no other cognitive advantage. Similarly, unpublished student research has revealed no other unusual psychological characteristics to suggest they are not representative of the wider population. Further details concerning the study survey are provided in Satchall et al. [21].

Volunteers are informed that most projects involve face recognition; however, they may also occasionally receive invites to diverse projects such as those measuring alcohol use and jury decision-making. Approval for creating the database was secured from the University of Greenwich Research Ethics Committee, and procedures to protect privacy follow GDPR and UK data protection laws. For the current research, approximately 12,000 participants from the UK were invited by e-mail. This included a brief description of the project, ethical and database withdrawal information, and a URL link to the research questionnaires loaded on the Qualtrics.com survey site (accessed 13th July 2020).

### 2.1. Survey Data

The following types of demographic data were collected: gender, age, ethnicity, highest education level and gross income. There were also a series of questions relating to whether alcohol consumption changed during the lockdown period and patterns of home drinking (alone or with other household members) and alcohol purchasing (online, supermarkets or for parties). 

### 2.2. Non-Sociodemographic Study Measures

#### 2.2.1. AUDIT-C

The AUDIT-C uses the first three consumption items of the Alcohol Use Disorders Identification Test (AUDIT) [13]. The three AUDIT-C items relate to frequency of consumption (never–4+ times per week), amount consumed in units on a typical day when drinking (1–10 units) and how often an individual consumed 6 or more units if female or 8 or more if male on a single occasion in the last year. The range of scores is 0–12. Higher scores indicate greater consumption. This paper investigates predictors of at-risk drinking using different cut-off scores. These are shown in Table 2.

Participants were given pictorial representations of drinks and were told that a drink (1 unit) equates to half a UK pint of beer (approximately 500 ml), 175 ml of normal-strength wine and one UK measure of spirits (25 ml). Higgins Biddle and Babor [22] recommend that when using the AUDIT-C as a screening tool, this information should be provided to assist the participants. The psychometric properties of the AUDIT-C have been established in general population studies in the US (*n* = 26,946) [23] and more recently, in Korea (*n* = 222) [24].

#### 2.2.2. Home Drinking Assessment Scale (HDAS)

Foster et al. [12] described the design and testing and factor structure of the HDAS. It consists of 9 items and has a Cronbach’s alpha of 0.83. The same paper presents the internal consistency for each factor. Factor 1 (emotional reasons) produced an alpha of 0.73, and Factor 2 (practical reasons) an alpha of 0.44. The overall alpha for the scale was 0.61. The items are divided into the following motivational reasons for drinking at home: emotional reasons (5 items) (e.g., drinking at home because it was safer than going out) and practical reasons (3 items) (e.g., the cost of alcohol in pubs/bars/restaurants). There is one additional item: “I prefer to drink alcohol at home rather than a pub/restaurant etc.”. Each item is scored on a scale of 1 (strongly agree with statement) to 5 (strongly disagree with the statement). In the current research, Cronbach’s alpha = 0.787, which is indicative of acceptable reliability [25].

#### 2.2.3. Additional Home Drinking Items

These items were originally derived from Foster and Canfield [11]. Five questions were asked concerning the context in which alcohol is consumed at home. Two of these were condensed in two themes. Firstly, “With meals”: (a) consuming alcohol with meals, (b) at a barbeque or similar. Secondly, “With entertainment”, which comprises (a) watching television/downloads, (b) playing on screen games/social media and finally, (c) reading books or newspapers. The scoring is the same as that described for the HDAS.

#### 2.2.4. Changes in Alcohol Consumption

A question was added concerning whether there was a change in alcohol consumption compared to prior the lockdown. Less than before was scored 1, the same 2 and more than before 3.

#### 2.2.5. Purchasing Behaviour

A series of questions were asked concerning purchasing alcohol before and after the lockdown period. The areas of enquiry were purchasing alcohol at a supermarket, at an off licence (smaller shop selling alcohol), for parties and online. Participants were asked to indicate the level of agreement with whether they engaged in purchasing behaviour. Not applicable was scored 0, and thereafter there was a five-point scale: strongly agree (1)–strongly disagree (5).

#### 2.2.6. WEMWBS

Mental health and well-being were assessed by the Warwick and Edinburgh Mental Health and Well-being Scale (WEMWBS) [26]. This is a measure that can be used with the general population, and its validity and reliability is well established, e.g., [27]. The version used in the current study had 14 items, and there were five possible responses (scores in brackets): none of the time (1), rarely (2), some of the time (3), often (4), and all of the time (5). Thus, higher scores indicated positive mental health and well-being. The scale has a one-factor solution [28], and the items in the scale include questions concerning how optimistic an individual is about their future and how interested they are in new things. A score of below 40 indicates “probable depression”, 41–44 indicates possible depression, scores of 45–59 indicate average mental health well-being and scores of 60 or above are high mental health and well-being [29]. In the current research, the Cronbach’s alpha = 0.924, which was indicative of excellent reliability [25].

### 2.3. Data Analysis

Initially, descriptive data were presented. Thereafter, in order to construct final regression models, all the study variables were tested against all three cut-offs using a univariate analysis. Pearson’s product moment correlation co-efficient was computed to test for multicollinearity. If there was an association beyond the 0.8 level, in accordance with a large effect [25], then further univariate tests were computed to see which of the two had the largest effect. The one with the smallest t statistic was excluded from the regression. Any variables that passed the cut-off of *p* ≤ 0.05 in any of the three univariate analyses were entered into final regression model as independent variables. These were three hierarchical logistic regressions based on the findings of Foster and Canfield [11]. The dependent variable on each occasion was the relevant at-risk drinking cut-off. Beta statistics, odds ratios, 95% CIs and probability values are reported.

## 3. Results

One thousand six hundred and twenty-eight participants clicked on the survey link, and of these, 138 did not start the survey and 312 did not complete it. A further fifty were excluded as they did not complete one of the main study measures, the HDAS. The characteristics of the sample (*n* = 1128) are shown in Table 1. Over seventy percent of the sample were women, and the mean age of the sample was 45.05 (12.3 SD). Over ninety-five percent described themselves as white. This was a highly educated sample with (*n* = 648, 57.4%) being educated to an undergraduate level or above. Over half of the sample of those who replied had monthly incomes of less than £2000. The mean AUDIT-C score was 6.93 (SD = 2.40). This is above the cut-off recommended by Public Health England [16]. The AUDIT-C data will be discussed in greater depth shortly. Nearly seventy percent (*n* = 794, 69.6%) of the participants were either drinking the same or less than before the lockdown period. The mean amount of money spent per month on alcohol during the lockdown was £24.6 (SD = 255.6). The WEMWBS scores were at the bottom range of average mental health and well-being.

### 3.1. Reasons for Drinking at Home

All participants responded to each HDAS item. For the practical reasons subscale, cost was the most endorsed motivation (mean = 2.88, SD = 1.35), followed by, in descending order, not having to drink and drive (mean = 3.15, SD = 1.46), not feeling comfortable drinking out (mean = 3.95, SD = 1.15), because they had children and could not go out (mean = 4.18, SD = 1.16) and lastly, not being able to smoke in licensed premises (mean = 4.66, SD = 0.73). With regards to the emotional reasons subscale, convenience was the most endorsed motivation (mean = 2.91, SD = 1.24), followed by relaxation (mean = 3.05, SD = 1.27) and drinking at home because it was safer than going out. (mean = 3.30, SD = 1.26). The statistics for the HDAS stand-alone item “I prefer to drink at home rather than a pub or restaurant” were (mean = 3.22, SD = 1.22).

In addition to the HDAS questions, five questions asked about activities conducted whilst consuming alcohol during the lockdown period. They were scored identically to the HDAS items, so that lower scores indicated higher levels of endorsement. The statistics for the “with food items” were having a barbeque in the garden (with members of the same household) (mean = 1.77, SD = 1.33) and with meals (mean = 2.19, SD = 1.34). The “with entertainment” items were endorsed less often than the items with food variable were. The statistics for “with entertainment” were (*n* = 1119) (mean = 7.97, SD = 4.44). These were subdivided into the following three items: watching TV (2.34, 1.49, Mean SD), watching or playing computer games or other screen activities. (mean = 2.60, SD = 1.99) and lastly, reading a book or a newspaper (mean = 3.02, SD = 1.79). There were also two questions (scored as above, so lower scores indicate greater frequency) relating to whom participants drank with during the lockdown period. Drinking with other members of the household was more common than drinking alone.

### 3.2. AUDIT Data and Gender Differences

The mean AUDIT-C score was 6.93, (SD = 2.40) (Table 1). Men had higher AUDIT-C scores (two-tailed Mann–Whitney U test) (men: *n* = 308, mean = 7.57, median = 7.00, SD = 2.63, range = 3–12) (women: *n* = 776, mean = 6.66, median = 6.00, SD = 2.23, range = 3–12) (U = 37.41, *p* < 0.001). Table 2 presents details for the number of participants who were AUDIT-C-positive using the different cut-off points. The only cut-off point where there was a significant between-gender difference was when the score was eight for both genders. Men were more likely to be AUDIT-C-positive than women were (men: *n* = 151, 49.0%) (women: *n* = 228, 29.3%) (chi-square = 37.97, df = 2, *p* < 0.001). All other between-gender differences were *p* > 0.4.

### 3.3. Gender Differences in Non-AUDIT-C Variables

There were no between-gender differences in age (*p* > 0.5). Women more likely to have a postgraduate qualification (males: *n* = 63, 19.2%; females: *n* = 263, 32.8%) (chi-square= 19.59, df = 7, *p* = 0.007). Men were more likely to have higher salaries (*p* = 0.004) with (*n* = 183, 57.7%) men having salaries £2000 or more compared to the equivalent figures for women (*n* = 352, 45.1%).

There were a number of questions concerning whether drinking had decreased, remained the same or increased during the lockdown period. There were no between-gender differences (*p* > 0.244) in any of these variables. Similarly, there were no gender differences in the typical weekly spend (£) on alcohol either before (*p* = 0.09) or during (*p* = 0.584) lockdown.

With regards to motivations for drinking (HDAS), there were no significant between-gender differences for both subscales (*p* > 0.4 on both occasions). There were no gender differences for the “with food” variable (*p* = 0.263); however, men were more likely to consume alcohol along with entertainment (males: *n* = 319, mean = 7.45, SD = 4.27; females: *n* = 794, mean = 8.18, SD = 4.50) ((−) t = 2.47, *p* = 0.014).

Women were more likely to state that purchasing for parties prior to the lockdown was the main reason for them purchasing alcohol to be consumed outside of pubs and bars (two-tailed independent sample t-test) (men: *n* = 321, mean = 2.62, SD = 1.27; women: *n* = 788, mean = 2.35, SD = 1.10) (t = 3.52, *p* < 0.001). During the lockdown period, the gender difference was no longer significant (*p* = 0.079). Prior to lockdown, there was no between-gender difference (*p* = 0.313), but during lockdown men were more likely to purchase alcohol as part of normal household shopping (men: *n* = 322, mean = 2.20, SD = 1.31; women: *n* = 795, mean = 2.45, SD = 1.40) (t= (−) 2.69, *p* = 0.007). There was no difference in the probability of men or women purchasing alcohol online either before or during the lockdown period (*p* > 0.2 on both occasions). There was no gender difference in drinking with members of the household (*p* = 0.678), but men were more likely to drink alone during the lockdown (men: *n* = 321, mean = 2.65, SD = 1.67; women: *n* = 793, mean = 2.94, SD = 1.72) (t= (−) 2.52, *p* = 0.012)

Finally, there were no gender differences (*p* > 0.5) in WEMBWS scores.

### 3.4. Age Differences and Testing for Multicollinearity

Data relating to age were collected as a continuous variable, and the relationships between age and other continuous variables collected are shown in Table 3. All of the coefficients had weak effects [25] (all ≤ 181). There were some significant relationships. Older age was significantly associated with higher (better outcomes) health and well-being scores (WEMWBS), and a greater likelihood of purchasing alcohol for parties during the lockdown period. The HDAS subscales showed results in differing directions. Older age had a negative relationship with practical motivations for drinking at home, whilst the relationship between age and emotional motivations for drinking at home was positive. There was little evidence of multicollinearity. All correlations were ≤0.565, so none met the cut-off for a large effect, which is r = 0.8 [25].

### 3.5. Comparison of Purchasing Behaviours during Lockdown

This data are is presented in Table 1. There was little difference in the monies spent per week on alcohol prior to lockdown and that spent in a typical week during lockdown (*p* = 0.802). Prior to the lockdown, the most common purchasing motivation was special occasions such as parties. During the lockdown period, this was less frequent, as indicated by higher scores. This was a significant change (t= (−) 24.72, *p* < 0.001). The next most prevalent purchasing pattern prior to the lockdown was as part of normal household shopping. This was more prevalent during lockdown. The within-group difference was significant (t = 10.22, *p* < 0.001).

Purchasing alcohol in an off licence before the lockdown was infrequent. Data could not be collected relating to this during lockdown due to restrictions on the opening of such outlets. Purchasing alcohol online prior to the lockdown was infrequent and was significantly more popular during lockdown (*n* = 1107) (mean = 3.68, SD = 1.49) (t = 6.64, *p* < 0.001).

### 3.6. Univariate Testing at Different AUDIT-C Cut-Offs

Two demographic variables were not related to any of the designated cut-off points, these were age (*p* > 0.6), and educational level (*p* > 0.2). Changes in the amount spent on alcohol during the lockdown compared to before lockdown (*p* > 0.1), “with meals” (*p* > 0.19) or WEMBS scores (*p* > 0.1) were also not significant at any point. No further data are reported related to these variables.

### 3.7. Men 5/Women 5 (Public Health England Recommended Cut-Off)

There were (*n* = 909, 83.9%) participants who were AUDIT-C-positive using the Public Health England recommended cut-off. The relationships with the cut-off score and gender (*p* = 0.235) and household status (*p* = 0.197) were not statistically significant. The association with income was significant with 86% of the sample who had a monthly salary of £2000 or more reaching the cut-off score for an at-risk drinker (chi-square = 16.7, df = 4, *p* = 0.002). The relationships with either drinking with members of the household (*p* > 0.9) or drinking alone (*p* > 0.35) were not significant.

Drinking more than before lockdown was associated with the at-risk drinking (*n* = 316, 96.9%) and being AUDIT-C-positive (chi-square = 58.4, df = 2, *p* < 0.001). The same was true for those who spent more on alcohol during than lockdown than before (*n* = 249; *n* = 240, 96.3%); they were AUDIT-C-positive (chi-square = 36.9, df = 2, *p* < 0.001). All purchasing patterns during lockdown were associated with at-risk drinking at a *p* < 0.001 level. The most common purchasing pattern was firstly, at a supermarket (AUDIT-C+: mean = 2.09, SD = 1.18; AUDIT-C−: mean = 3.41, SD = 1.52), secondly, for a special occasion such as a party (AUDIT-C+: mean = 3.40, SD = 1.41; AUDIT-C−: mean = 3.85, SD = 1.36) and lastly, online (AUDIT-C+: mean = 3.50, SD = 1.52; AUDIT-C−: mean = 4.42, SD = 1.05).

Both HDAS subscales were related with the at-risk drinking cut-offs. The statistics were as follows: emotional reasons (AUDIT-C+: mean = 8.78, SD = 2.74; AUDIT-C−: mean = 10.90, SD = 3.02) (t = (−) 9.19, df = 1088, *p* < 0.001); practical reasons (AUDIT-C+: mean = 18.51, SD = 3.60; AUDIT-C−: mean = 19.72, SD = 3.99) (t = (−) 4.02, df = 1088, *p* < 0.001).

There was an inverse relationship in the “with entertainment” variable (AUDIT-C+: *n* = 908, mean = 8.41, SD = 3.94) (AUDIT-C−: *n* = 174, mean = 7.08, SD = 5.71) (t = 2.93, *p* = 0.004) (lower scores indicate greater endorsement). “Drinking whilst reading a book or newspaper” had the strongest relationship to at-risk drinking (AUDIT-C+: mean = 3.21, SD = 1.63) (AUDIT-C−: mean = 2.61, SD = 2.14) (t = 4.18, *p* < 0.001). “Playing computer games or other screen activities” was also significant, albeit at a slightly lower level (AUDIT-C+: mean = 2.75, SD = 1.92) (AUDIT-C−: mean = 2.27, SD = 2.19) (t = 2.94, *p* = 0.008). Both were inverse relationships. The probability level of the “Watching TV” variable was (*p* = 0.064).

### 3.8. Men 8/Women 7: Student and Young People Cut-Off

There were (*n* = 515, 47.9%) participants who were AUDIT-C-positive at this cut-off point. Once more, there were no significant associations between this cut-off and gender (*p* = 0.839) or income (*p* = 0.063).

Drinking more than before lockdown was associated with the at-risk cut-off, (*n* = 235, 72.5%) (chi-square = 115.0, df = 2, *p* < 0.001). The same was true for those who spent more on alcohol during than lockdown than before. One hundred and eighty-six (75.0%) participants who spent more on alcohol during lockdown than previously were AUDIT-C-positive (chi-square= 93.49, df = 2, *p* < 0.001).

Once more, all three of the purchasing category variables were associated with being AUDIT-C+ at a *p* < 0.001 level. The hierarchy was as before (in descending order): “at a supermarket” (AUDIT-C+: mean = 1.90, SD = 1.08) (AUDIT-C−: mean = 2.68, SD = 1.43), for special occasions such as parties (AUDIT-C+: mean = 3.26, SD = 1.41) (AUDIT-C−: mean = 3.67, SD = 1.37) and finally, online (AUDIT-C+: mean = 3.26, SD = 1.56) (AUDIT-C−: mean = 4.01, SD = 1.32).

Both HDAS subscales were related with the at-risk drinking cut-offs, though the relationship with emotional reasons had the strongest relationship. The statistics were as follows: emotional reasons (AUDIT-C+: mean = 8.34, SD = 2.64) (AUDIT-C−: mean = 9.88, SD = 2.92) ( t= (−) 9.03, df = 1079, *p* < 0.001.) Practical reasons (AUDIT-C+: mean = 18.48, SD = 3.71) (AUDIT-C−: mean = 18.92, SD = 3.67) (t= (−) 1.96, df = 1079, *p* = 0.050) The relationships “with entertainment” and with meals were not significant (*p* > 0.5).

There was a positive significant relationship with both drinking with members of the household (AUDIT-C+: mean = 1.62, SD = 1.07) (AUDIT-C−: mean = 1.89, SD = 1.38) (*p* < 0.001) and drinking alone (AUDIT-C+: mean = 2.80, SD = 1.55) (AUDIT-C−: mean = 3.04, SD = 1.77) (*p* = 0.020).

### 3.9. Men 8/Women 8: Cut-Off for Adults Seeking Online Help

There were (*n* = 379, 34.9%) participants who were at-risk drinkers according to this cut-off value. On this occasion, men were more likely to be at-risk drinkers; men (*n* = 151, 49.0%), women (*n* = 228, 29.3%) (Men: Women) (chi-square = 37.41, df = 1, *p* < 0.001). Income was also significantly related to at-risk drinking (chi-square = 10.5, df = 4, *p* = 0.032). Those with a salary per month of more than £2000 (*n* = 199, 38.1%) were more likely to be at-risk drinkers than were those with a lower salary (*n* = 179, 31.2%).

Drinking more than before lockdown was associated with the at-risk cut-off, (*n* = 180, 55.0%) (chi-square = 87.2, df = 2, *p* < 0.001). The same was true for those who spent more on alcohol during than lockdown than before (*n* = 143, 57.4%) (chi-square = 70.86, df = 2, *p* < 0.001).

Once again, all the purchasing variables were significant at a *p* < 0.001 level. The hierarchy was as follows: (in descending order): (a) at a supermarket (mean = 1.83; SD = 1.03), (b) for special occasions such as parties (mean = 3.14; SD = 1.42) and (c) online (mean = 3.18; SD = 1.55).

There was a positive significant relationship with both drinking with members of the household (AUDIT-C+: mean = 1.64, SD = 1.08) (AUDIT-C−: mean = 2.74, SD = 1.53) (*p* = 0.020) and drinking alone (AUDIT-C+: mean = 2.74, SD = 1.53) (AUDIT-C−: mean = 3.02, SD = 1.73) (*p* = 0.008).

Both HDAS subscales were related with the at-risk drinking cut-offs, though the relationship with emotional reasons had the strongest relationship. There were (*n* = 382, 35.1%) who were AUDIT-C-positive. The statistics were as follows: emotional reasons (AUDIT-C+: mean = 8.20, SD = 2.56) (AUDIT-C−: mean = 9.62, SD = 2.94) (t= (−) 7.94, df = 1088, *p* < 0.001); practical reasons (AUDIT-C+: mean = 18.34, SD = 3.81) (AUDIT-C−: mean = 18.91, SD = 3.61) (t= (−) 2.42, df = 1079, *p* < 0.016). The association between this cut-off point and “with entertainment” was of borderline statistical significance (*p* = 0.054).

### 3.10. Regression Models

The univariate results were used to assemble a logistic regression model that allowed the predictors of at-risk drinking at all three cut-offs, shown in Table 2, to be established. The following variables were entered into stepwise regression analyses: (a) demographic variables, gender and income, (b) purchasing of alcohol during lockdown—online, supermarkets or for parties, (c) drinking alcohol alone or with other members of the household, (d) HDAS emotional and practical reasons scores and (e) “with entertainment”.

### 3.11. Results of Heirarchical Regressions

The results of the hierarchical regressions at the three cut-offs are shown in Table 4 and Table 5. The hierarchy was based upon the findings of Foster and Canfield [11] and was as follows for Table 4 (highest first): (a) purchasing patterns during lockdown, (b) context of drinking, (c) motivations for drinking at home and (d) sociodemographic variables. It was expected there would come a point where gender would be a significant predictor, so for Table 5, gender is entered first in the regression; income was entered last. Otherwise, the ordering for Table 5 is the same as for Table 4. Gender was not significant until the final regression; at that point, men were more likely to be at risk drinkers than women were. Income was not significant at any point. Purchasing alcohol online and at a supermarket was associated with at-risk drinking at all three cut-off points. Buying alcohol for parties was not significant at the lowest cut-off point but was significant thereafter. Similarly, at the lowest cut-offs, the drinking contexts were not significant, but both drinking with members of my household and alone were significant predictors at the higher cut-off levels. Emotional motivations (HDAS) for drinking at home were significant on all three cut-off points. Practical motivations were not significant at the lower cut-off points, but again became significant at the two higher cut-offs. The emotional reasons score shows a negative relationship, which means that greater endorsement of emotional reasons was significantly related to at-risk drinking but the practical reasons presented a negative relationship. These findings were consistent across all three cut-off points. Drinking “with entertainment” was significant at lower cut-off levels, but significance was lost at higher cut-offs.

## 4. Discussion

This paper collected data concerning alcohol consumption during a government-directed COVID-19 lockdown period in which pubs and restaurants were closed. Therefore, it was possible to focus upon drinking at home and accompanying purchasing patterns. The focus was upon predictors of at-risk drinking using three established cut-off points of the AUDIT-C. Applying the limits suggested by Public Health England [16] (a score of 5 for both genders), the predictors of at-risk drinking were purchasing alcohol online, supermarket shopping, emotional reasons for drinking at home and drinking “with entertainment”. When the cut-off point for students and young people (Females 7, Males 8) [18] was used, the predictors were emotional reasons but not and practical reasons for drinking at home, purchasing alcohol in a supermarket, online or for parties and drinking with members of the household and drinking alone. Finally, a cut off 8 for both genders was tested, which has been established for adults seeking online help because of their alcohol consumption [19]. The predictors were similar to those for the student and young people’s cut-offs, although in this analysis, gender was significant. Age, educational level and health and well-being scores were not related to at-risk drinking scores.

When considering the cut-off recommended by Public Health England, the significant predictors in this study were purchasing alcohol online, purchasing alcohol at a supermarket, emotional drivers for drinking at home and drinking “with entertainment”. It is informative to compare these results with those of Foster and Canfield [11]. In that paper, the cut-off for at-risk drinking was based on the total AUDIT score [13], and the significant predictors of at-risk drinking in the home revealed in their study were being female, younger in age and drinking alcohol at home because it is cheaper than drinking out. The “with entertainment” variable consisted of drinking alcohol with a combination of watching television, onscreen activities and/or reading a book or newspaper. This was significantly related to at-risk drinking at the lowest cut-off but not at higher levels. The reasons for this are unclear and indicate an avenue for future investigation.

There were more significant predictors of at-risk drinking when the cut-off was that tested on students and young people (7 females, 8 males) [18]. These were firstly, emotional reasons for drinking at home and practical reasons for drinking at home, and secondly, purchasing alcohol in a supermarket, online, and for parties and drinking with members of the household, and drinking alone. This indicates that when it is not possible to drink in a pub and bar, motivational reasons, purchasing patterns and situational factors combine to explain at-risk drinking in a domestic situation. Gender was not significant in this analysis, suggesting that there is little difference in the context of home drinking between men and women during the lockdown period.

The final cut-off was 8 for both genders, which has been established as a cut-off for adults seeking online help as a consequence of their drinking [14]. On this occasion, the significant predictors were the same as previously, with two exceptions. At this point, gender was significant with men being more likely to be at-risk drinkers. In contrast, the “with entertainment” variable was not significant. The emotional and practical motivations for drinking at home were in differing directions (as was the case in each regression). The relationship between emotional reasons was negative, meaning that greater endorsement of emotional reasons for drinking at home was associated with greater likelihood of at-risk drinking. The motivations relating to practical reasons for drinking at home were in the opposite direction.

### 4.1. Demographic and Contextual Determinants of At-Risk Drinking

In the current study, gender was only significant at the highest cut-off point when men were more likely to be at-risk drinkers. This suggests that when the opportunities to drink in pubs and bars are curtailed, there is little gender difference in alcohol consumption patterns at lower at-risk cut-offs. The fact that men are drinking at a higher level is consistent with much research [30], although the gap between genders is narrowing [31].

The relationship between age and the study variables (i.e., effect size) was very weak. There were however some significant findings. Older age was related with better health and well-being skills and a greater likelihood of purchasing alcohol for parties during lockdown. Older age was also associated with endorsing emotional reasons for drinking at home and not selecting practical reasons. The findings in our study concerning gender and age are consistent with Spanish data collected during the lockdown period [32].

None of the measures in the study that could approximate to social class, income and highest education level were associated with any of the at-risk cut-offs. This is contrary to findings from the Alcohol Toolkit Study (*n* = 57,807) [33], which found that “social grade” and highest educational level were the strongest predictors of general population alcohol consumption. Beard et al. [33] collected data prior to the lockdown in 2014–2018 and also found that housing status was a key predictor of alcohol consumption; in comparison, employment status and car ownership were poor predictors. Villanueva et al. [32] found that individuals who were self-employed were more likely to drink at an at-risk level than were full-time employees. In the current study, no data were collected concerning employment status.

### 4.2. Summary of AUDIT-C Cut Off Findings

Emotional reasons for drinking at home was a significant predictor at all three cut-off points. This means that convenience, relaxation and drinking at home because it is safer than going out were drivers for at-risk drinking at all levels. In contrast, practical reasons was not a significant factor until the two highest cut-offs and was in a different direction to emotional reasons. This means that as at-risk drinking increases, issues such as cost, drinking and driving, not feeling comfortable going out, child care and smoking are weak explanations of at-risk drinking.

Purchasing alcohol online and at a supermarket was a significant factor at all three cut-off points, whereas purchasing alcohol for parties was only significant at the two higher cut-offs. This indicates that this could be a marker for higher at-risk drinking both during and outside of lockdown. Drinking alone and as part of a household were not significant at the lowest cut-off, but both variables were significant at higher cut offs. All these suggest that as drinking opportunities increase, then the likelihood of at-risk drinking increases also.

Men had higher AUDIT-C scores, but gender was not significant until both genders had AUDIT-C scores of 8. This does suggest that further work needs to be carried out to establish whether the behaviours that have been associated with at-risk drinking during the lockdown period are maintained when pub/bar and restaurant drinking return to pre-lockdown patterns. Then, it could be beneficial to revisit the AUDIT-C cut-offs for each gender. One possibility could be to establish a base at-risk cut-off and a higher one indicative of higher-risk drinking. This could then be a prompt to offer referral to lower-threshold treatment services. These findings suggest the cut-off for higher-risk drinking would be 8 for women.

The COVID-19 lockdown period was exceptional; however, these findings indicate that home drinking has become a habitual driver of alcohol consumption. This may be an important consideration for both treatment providers and policymakers, albeit a longitudinal study with a follow-up period of one year or more would be required to see if these changes have become entrenched. We suggest that the HDAS (9 items) is routinely integrated into research that is examining the trajectory of both at-risk and dependent drinkers. The findings are of import as women with this age profile (mean age of 45) have received little research focus in alcohol studies. The sample was highly educated, but 45% of the women had a net income more than £2000 per month. They confirm that home drinking is a driver of at-risk drinking. Eighty-five per cent of the sample reported that they were at-risk drinkers when the recommended Public Health England limits were applied, and nearly half of the sample were at-risk drinkers when a limit for student and young people was used. On both occasions, there was no between gender differences. It was only when the cut-off for seeking online help was tested (AUDIT-C scores of 8 for both genders) that males were more likely to be at-risk drinkers.

Cost was the most frequently motivation for drinking at home. Scotland introduced a policy of minimum unit pricing (MUP) in May 2018, and Wales followed suit in March 2020. The Republic of Ireland will introduce a similar policy in January 2022. This means that in Scotland and Wales, the minimum unit price for a unit of alcohol is 50 p. Robinson et al. [34] found that MUP was associated with a fall of 2% in Scottish off sales in comparison with an equivalent rise of 2.4% in England and Wales (at this point Wales had not introduced MUP) in the first year MUP was introduced in Scotland. The beverages associated with the fall in consumption in Scotland were spirits and ciders/perry-based beverages. Anderson et al. [35] also considered early data from Wales as well as Scotland. In both cases, there was a positive impact from MUP. Alcohol consumption fell, and the greatest impact of MUP was on those families who consumed/purchased the most alcohol. There have recently been calls in Scotland to raise the MUP to 65 p per unit to take account of inflation [36] (Alcohol Focus Scotland 2021). Currently, there are no plans to introduce MUP in England.

### 4.3. Strengths and Limitations

Despite the rise of home drinking, it has received comparatively little research attention. The current research employed a relatively large participant sample, and most of the sample are women (mean age of the sample 45+). The analysis has included an investigation of the association between home drinking and health and wellbeing. However, there are some limitations that should be acknowledged. Firstly, this is a cross-sectional study, and no data were collected before the lockdown period, so no conclusions concerning causation can be inferred. Data were not collected concerning employment status or whether individuals were working during the lockdown period, either at home or outside the home, and these may have had an impact on the study findings. Secondly, this is an internet survey, and whilst these are increasing common, despite the sample size it is not possible to draw conclusions about representativeness of the sample. Asking questions concerning alcohol is regarded as sensitive, and women are more likely to respond to online surveys than men are [37]. Even though there was a high proportion of the sample who reported at-risk drinking, research suggests there are several reasons that indicate consumption levels are under-reported. Boniface and Shelton [38] compared alcohol consumption data from the Health Survey for England and the General Lifestyle Survey and found systematic under-reporting of self-reported consumption. Other research has confirmed that individuals tend to use their own experiences to equate how their drinks correspond to units of alcohol [39], and there is also the issue of accurate recall. Finally, when estimating wine consumption, there is a tendency to underestimate the strength and amount of the drink being served or poured [40]. Wine is the drink of choice for women, and in this sample, 70% of the sample were women.

On the other hand, it should be acknowledged that this proclivity to display high drinking patterns may be a property of the specific sample of participants recruited who are interested in face recognition research and mainly possess far better than average face recognition ability. The subset of participants invited who completed the survey may have found the topic of alcohol use during lockdown particularly salient, and their results may not be representative of the rest of the database, let alone the wider population. Nevertheless, public health research of this type rarely assesses participants with this demographic profile, and follow-up research is required to determine if lockdown has established a pattern of at-risk drinking in a demographic not previously identified as such.

## 5. Conclusions

Notwithstanding that a lockdown is not a typical drinking culture, this paper confirms that home drinking is an integral part of the current drinking culture. A large proportion of the sample were women, and the mean age was 45. Over eighty percent of the sample were at-risk drinkers using current Public Health England recommended cut-offs, and nearly half of the sample were at-risk drinkers when a cut-off used for young people and student was used. The predictors of at-risk drinking at home were a combination of motivational reasons, purchasing patterns and situational factors. The Home Drinking Assessment Scale has produced some valuable data, and future researchers should consider including it to understand how home drinking contributes to a trajectory of harmful, hazardous and dependent drinking.

## Figures and Tables

**Table 1:** Characteristics of the total sample (*n* =1128).

	Numbers	%
Gender		
Males	322	28.5
Females	838	71.5.
No Response	6	<1%
Ethnicity		
White	1075	95.3
Others	53	4.7
Highest Educational Level		
No Exams	14	1.2
Other	31	2.7
GCSE Only	113	10.0
A Level Equivalent	141	12.5
Further Education or Equivalent	181	16.0
Undergraduate Degree	381	33.8
Masters Degree	235	20.8
Doctorate	32	2.8
Pre-Tax Monthly Income. (*n* = 1102)		
Less than £2000	561	50.9
£2000 or more	541	49.1
Drinking Patterns during Lockdown (*n* = 1120)		
Less than Before	334	29.8
About the Same	460	41.1
More than Before	326	29.1
	Mean	SD
Age	45.05	12.3
Typical Weekly Spend on Alcohol (£s) (*n* = 1105)		
Before Lockdown	23.4	168.7
During Lockdown	24.6	255.6
HDAS Scores		
Practical Reasons	18.81	3.73
Emotional Reasons	9.26	2.97
Other Reasons for Drinking at Home		
With Food (*n* = 1124)	3.95	2.18
With Entertainments (*n* = 1119)	7.97	4.44
Purchasing Behaviour Prior to Lockdown		
For Parties (*n* = 1115)	2.43	1.16
Normal Household Shopping (*n* = 1118)	2.85	1.38
From an Off Licence (*n* = 1103)	3.89	1.13
Online (*n* = 1107)	3.93	1.20
Purchasing Behaviour during the lockdown		
For Parties (*n* = 1117)	3.52	1.41
Normal Household Shopping (*n* = 1123)	2.38	1.39
Online (*n* = 1119)	3.68	1.48
Drinking Contexts During the Lockdown		
Other Members of the Household (*n* = 1117)	1.73	1.27
Drinking Alone (*n* = 1120)	2.85	1.71
WEMWBS (*n* = 1128)	45.4	9.41
AUDIT-C (*n* = 1090)	6.93	2.40

**Table 2:** AUDIT-C cut-offs used in this study and gender differences.

Audit-C Cut-Offs	AUDIT-C Positive		AUDIT-C Negative	
	Number	%	Number	%
Public Health England recommended cut-off: Males and Females both ≥5 [16] (*n* = 1084)	909	83.8	175	16.2
Males (*n* = 308)	265	86.0	43	14.0
Females (*n* = 776)	644	82.9	132	17.1
Students and young people Females ≥7 Males ≥8 [18] (*n* = 1075)	515	47.9	560	52.1
Males (*n* = 307)	149	48.5	158	51.5
Females (*n* = 768)	366	47.6	402	52.3
Adults seeking on-line help ≥8 Both genders [19] (*n* = 1084)	379	34.9	705	65.1
Males *** (*n* = 308)	151	49.0	157	51.0
Females (*n* = 776)	228	29.3	548	70.7

*** *p* < 0.001.

**Table 3:** Correlations of continuous study variables during lockdown.

	Age	Alcohol Spend	WEWMBS	Audit-C	HDAS-E	HDAS-*p*	Meals	Entert	Onl	Super	Party
Age	1	0.002	0.181	0.020	−0.068	0.113	−0.035	−0.050	−0.034	0.015	0.174
Sig		0.994	0.001	0.518	0.022	<0.001	0.246	0.093	0.259	0.607	<0.001
Alcohol spend		1	0.060	0.019	0.041	0.015	−0.071	−0.066	−0.001	−0.045	−0.016
Sig			0.046	0.525	0.172	0.630	0.019	0.028	0.986	0.140	0.601
WEMBWS			1	−0.050	0.129	0.130	−0.054	0.005	0.049	0.004	−0.042
Sig				0.099	<0.001	<0.001	0.072	0.857	0.105	0.887	0.164
Audit-C				1	−0.319	−0.098	−0.013	−0.013	−280	−0.369	−0.189
Sig					<0.001	0.001	0.660	0.659	<0.001	<0.001	<0.001
HDAS-E					1	0.559	−0.109	−0.041	0.278	0.329	0.094
Sig						<0.001	<0.001	0.170	<0.001	<0.001	0.002
HDAS-*p*						1	−0.137	−0.094	0.172	0.198	0.129
Sig							<0.001	0.002	<0.001	<0.001	<0.001
Meals							1	0.565	−0.014	−0.185	−0.202
Sig								<0.001	0.645	<0.001	<0.001
Entert $								1	−0.73	−220	−179
Sig									0.014	<0.001	<0.001
Onl *									1	0.095	0.106
Sig										0.002	<0.001
Super **										1	0.330
Sig											<0.001
Party ***											1

The top row of each variable shows Pearson product moment correlation coefficients. Sig = Probability (*p*-value). HDAS-E: = HDAS emotional reasons subscale. HDAS-*p* = HDAS practical reasons subscale. $ Drinking “with entertainment”; * purchasing alcohol online during lockdown; ** purchasing alcohol at a supermarket during lockdown; *** purchasing alcohol for a party during lockdown.

**Table 4:** Hierarchical regression for Regression 1 (AUDIT-C cut-off 5 for both genders) and Regression 2 (AUDIT-C scores of 7 for females and 8 for males).

	Beta	Df	OR	95% Cis	*p*
Block One: Purchasing Patterns During Lockdown: Regression 1					
Purchasing alcohol online	(−) 0.415	1	0.660	0.561–0.777	<0.001
Purchasing alcohol in a supermarket	(−) 0.568	1	0.567	0.490–0.655	<0.001
Purchasing alcohol for parties	0.027	1	1.027	0.885–1.191	0.725
Regression 2					
Purchasing alcohol online	(−) 0.315	1	0.730	0.661–0.806	<0.001
Purchasing alcohol in a supermarket	(−) 0.474	1	0.622	0.549–0.706	<0.001
Purchasing alcohol for parties	(−) 0.140	1	0.869	0.784–0.964	0.008
Block Two: Context of Drinking: Regression 1					
Drinking with members of the household	(−) 0.070	1	0.932	0.806–1.079	0.347
Drinking alone	(−) 0.089	1	0.915	0.809–1.034	0.154
Regression 2					
Drinking with members of the household	(−) 0.185	1	0.831	0.739–0.934	0.002
Drinking alone	(−) 0.123	1	0.885	0.805–0.973	0.011
Block 3: Motivations for Drinking at Home: Regression 1					
HDAS Emotional Reasons	(−)0.140	1	0.890	0.801–0.944	<0.001
HDAS Practical Reasons	0.045	1	1.046	0.981–1.115	0.172
With Entertainment	0.049	1	1.050	1.004–1.099	0.033
Regression 2					
HDAS Emotional Reasons	(−) 0.170	1	0.844	0.794–0.897	<0.001
HDAS Practical Reasons	0.076	1	1.079	1.031–1.129	0.001
With Entertainment	(−) 0.005	1	0.995	0.958–1.033	0.792
Block 4: Sociodemographic Variables: Regression 1					
Gender	(−) 0.004	1	0.996	0.644–1.542	0.987
Income	0.073	1	1.076	0.921–1.256	0.356
Regression 2					
Gender	0.290	1	1.337	0.981–1.820	0.066
Income	0.106	1	1.111	0.933–1.244	0.067

**Table 5:** Hierarchical regression for Regression 3 (AUDIT-C cut off 8 for both genders).

	Beta	Df	OR	95% Cis	*p*
Block One: Gender:					
Gender	(−) 0.688	1	0.502	0.369–0.685	<0.001
Block Two: Purchasing Patterns During Lockdown:					
Purchasing alcohol online	(−) 0.280	1	0.756	0.685–0.834	<0.001
Purchasing alcohol in a supermarket	(−) 0.450	1	0.638	0.555–0.733	<0.001
Purchasing alcohol for parties	(−) 0.216	1	0.805	0.725–0.895	<0.001
Block Three: Context of Drinking:					
Drinking with members of the household	(−) 0.128	1	0.880	0.788–0.995	0.041
Drinking alone	(−) 0.114	1	0.893	0.809–0.985	0.023
Block Four: Motivations for Drinking at Home:					
HDAS Emotional Reasons	(−) 0.151	1	0.860	0.807–0.916	<0.001
HDAS Practical Reasons	0.049	1	1.050	1.003–1.100	0.038
With Entertainment	(−) 0.025	1	0.976	0.938–1.015	0.277
Block Five: Income					
Income	0.075	1	1.078	0.959–1.211	0.207
